# Current and Future Trends on Diagnosis and Prognosis of Glioblastoma: From Molecular Biology to Proteomics

**DOI:** 10.3390/cells8080863

**Published:** 2019-08-09

**Authors:** Artemiy S. Silantyev, Luca Falzone, Massimo Libra, Olga I. Gurina, Karina Sh. Kardashova, Taxiarchis K. Nikolouzakis, Alexander E. Nosyrev, Christopher W. Sutton, Panayiotis D. Mitsias, Aristides Tsatsakis

**Affiliations:** 1 N. I. Pirogov Russian National Medical University, Russian Federal Ministry of Health, 117997 Moscow, Russia; 2 Federal State Institution V. P. Serbsky Federal Medical Research Center of Psychiatry and Narcology National Scientific Research Center on Addictions of the Ministry of Healthcare of the Russian Federation, 119002 Moscow, Russia; 3 Department of Biomedical and Biotechnlogical Sciences, University of Catania, 95123 Catania, Italy; 4 Research Center for Prevention, Diagnosis and Treatment of Cancer, University of Catania, 95123 Catania, Italy; 5 Department of Fundamental and Applied Neurobiology, Serbsky National Research Center for Social and Forensic Psychiatry, Ministry of Health and Social Development of the Russian Federation, 119034 Moscow, Russia; 6 Laboratory of Anatomy-Histology-Embryology, Medical School of Heraklion, University of Crete, Voutes, 71110 Heraklion, Crete, Greece; 7 Federal State Autonomous Educational Institution of Higher Education I.M. Sechenov, First Moscow State Medical University of the Ministry of Healthcare of the Russian Federation (Sechenov University), 119048 Moscow, Russia; 8 Institute of Cancer Therapeutics, University of Bradford, West Yorkshire BD7 1DP, UK; 9 Department of Neurology, School of Medicine, University of Crete, 71110 Heraklion, Crete, Greece; 10 Department of Neurology, Henry Ford Hospital, Detroit, MI 48202, USA; 11 Centre of Toxicology Science and Research, Faculty of Medicine, University of Crete, 71003 Heraklion, Greece; 12 Department of Analytical Toxicology, Sechenov University, 119048 Moscow, Russia

**Keywords:** molecular biology, proteomics, metabolomics, glioblastoma, mass spectrometry, biomarkers, miRNAs, DNA, proteins

## Abstract

Glioblastoma multiforme is the most aggressive malignant tumor of the central nervous system. Due to the absence of effective pharmacological and surgical treatments, the identification of early diagnostic and prognostic biomarkers is of key importance to improve the survival rate of patients and to develop new personalized treatments. On these bases, the aim of this review article is to summarize the current knowledge regarding the application of molecular biology and proteomics techniques for the identification of novel biomarkers through the analysis of different biological samples obtained from glioblastoma patients, including DNA, microRNAs, proteins, small molecules, circulating tumor cells, extracellular vesicles, etc. Both benefits and pitfalls of molecular biology and proteomics analyses are discussed, including the different mass spectrometry-based analytical techniques, highlighting how these investigation strategies are powerful tools to study the biology of glioblastoma, as well as to develop advanced methods for the management of this pathology.

## 1. Introduction: Glioblastoma Multiforme

Glioblastoma multiforme is characterized by poor prognosis, low survival rates, and extremely limited opportunities for therapy. Malignant gliomas are the third leading cause of cancer death for people aged between 15 to 34, accounting for 2.5% of the global cancer death toll. Among gliomas, glioblastoma multiforme represents the 50%, with a maximum incidence in patients aged more than 65 years [1,2,3,4]. Due to the absence of effective surgical and medical treatments currently available for glioblastoma, an early diagnosis coupled with an accurate tumor classification is of key importance to select a personalized treatment [5,6].

Gliomas are tumors with neuroectodermal origin, showing a considerable variability in age of onset, grade of severity, histological features, and ability to progress, as well as to metastasize [7,8]. According to the WHO classification, astrocytomas are histologically and clinically classified into four types: Pilocytic astrocytoma, diffuse astrocytoma, anaplastic astrocytoma, and glioblastoma multiforme. Pilocytic astrocytoma and diffuse astrocytoma are characterized by a relatively low growth rate, while for anaplastic astrocytoma and glioblastoma multiforme by common uncontrolled proliferation, diffuse tissue penetration, and neurodegeneration [9,10,11]. In turn, glioblastomas are classified into three subtypes, depending on the status of the IDH gene mutation: Primary glioblastomas (IDH-wild-type), secondary glioblastomas (IDH-mutant), and unclassified glioblastomas (NOS) [12]. It is important to note that unclassified glioblastomas (NOS) do not belong to a specific glioblastoma category, given their diagnostic and genetic heterogeneity; for that reason, they cannot be classified within any other group [7].

### 1.1. Diagnosis and Treatment of Glioblastoma

One of the main problems of glioblastoma management is related to the lack of effective diagnostic strategies. Currently, the main diagnostic methods for the detection of gliomas rely on neurological tests and neuroimaging methods, performed when the disease is already at an advanced stage [13,14]. Late diagnosis of glioblastoma is mainly caused by the slow dissemination process typical of brain tumors, which allows structures to gradually adapt to both compression and deformation caused by the tumor mass. For this reason, even in the case of pronounced morphological signs of tumor penetration into brain tissue, clinical manifestations may be completely absent [15]. However, a major drawback comes in patients which make use of antiangiogenic drugs or chemo-radiotherapy, that can significantly deceive the results coming from neuroimaging analyses, thus making the follow-up even more difficult [16,17,18,19].

A typical treatment for glioblastoma involves surgical resection of the tumor mass, followed by radiotherapy and chemotherapy treatments. However, such therapies are often proved to be ineffective, given the high rate of relapse, general tumor resistance appearance over time, coupled with a serious neurological deterioration of the patient [20].

Regardless its radicality, the surgical resection of glioblastomas is often inadequate, given the frequent residual presence of microscopic foci, leading to relapse or even recurrence of the disease [21,22]. This is mainly due to their infiltrative growth, as well as their high proliferative abilities. However, numerous studies have highlighted the importance of maximizing tumor removal to increase life expectancy of glioblastoma multiforme (GBM) patients. In fact, it is of key importance to remove the tumor mass up to the borders with the healthy surrounding tissue, in order to have a beneficial effect on patient survival rate [23,24]. However, even radical tumor resection is not conclusive since it is often followed by a relapse of the disease [25,26]. These findings explain why glioblastoma is not a surgically treatable disease [27,28,29,30,31,32].

More recently, based on the results obtained with other tumors [33,34], new treatments based on nitric oxide-releasing HIV protease inhibitors have been administered to glioblastoma patients [35,36]. Other studies have shown that despite the high vascularization of glioblastoma, treatments with the anti-VEGF bevacizumab do not significantly improve patients’ overall survival [37]. Finally, although several clinical trials have characterized the usage of carmustine wafers implants (Gliadel, generated 20 years ago) following tumor resection as adjuvant therapy, their clinical application has remained low [38,39].

### 1.2. Localization of Glioblastoma

Glioblastoma development occurs in the trans-barrier space of the blood-brain barrier (BBB), which prevents the translocation of polarized and/or high-molecular-weight substances from the bloodstream towards the brain [20]. Disturbances in BBB function linked with glioma malignancies are often observed, significantly affecting peripheral blood detectable levels of tumor biomarkers [40]. The rapid growth of glioblastoma cells creates areas of local hypoxia, which triggers the process of angiogenesis [41]. In addition to enhanced angiogenesis, changes in the expression of proteins of the aquaporin family in the components of the BBB have been linked with the tumor progression of the glioblastoma [42,43,44,45]. During tumor-induced angiogenesis, neo-formed vases show an abnormal structure, lacking the specific barrier function of normal BBB blood vessels. Surprisingly, this effect is stronger in those high-grade gliomas lacking almost totally the BBB barrier functionality, and weaker in diffuse gliomas and low-grade gliomas [46,47,48]. Noteworthy, all gliomas, including glioblastoma, show intact BBB areas, especially at the periphery of the tumor, representing one of the main obstacles against their response to drug treatments [49,50].

### 1.3. Characteristic and Carcinogenesis of Glioblastoma

During the past 20 years, an increase in the number of patients diagnosed with glioblastoma has been observed. This increase can be attributed both to the improvement of diagnostic investigations for brain tumors, and to an actual higher GBM incidence due to various occupational and environmental risk factors which may increase the incidence of all tumors, including glioblastomas [51,52,53,54,55,56]. Even though it is difficult to make a correlation between brain tumors and the exposure to environmental or lifestyle factors, numerous risk factors have been described to predispose to gliomas and glioblastomas [57]. Several studies have demonstrated that the gut microbiota is also correlated with tumor development [58,59,60]. Recently, different reports have described the existence of a so called “gut-brain axis”, showing that the dysregulation of the gut microbiota may lead to the alterations of several processes predisposing to the development of a number of nervous system diseases, including cancer [61,62]. Moreover, individual genetic background may be linked to the prognosis of patients. Generally, Asian glioblastoma patients survive longer compared with Caucasian, African, or Latin American patients [63]. Glioblastoma is more common in adult patients, mainly affecting the cerebral hemispheres; much less common in children and, as rule, localized in the region of the brain stem [64]. Radiographic contrast enhancement brain tissues studies have revealed significant infiltrates of tumor cells outside the contrasted tumor. This observation provides incontrovertible evidence that a clinically significant tumor burden also exists outside the tumor volume, thereby supporting the classification of GBM as a whole brain disease [65].

**Figure 1:**
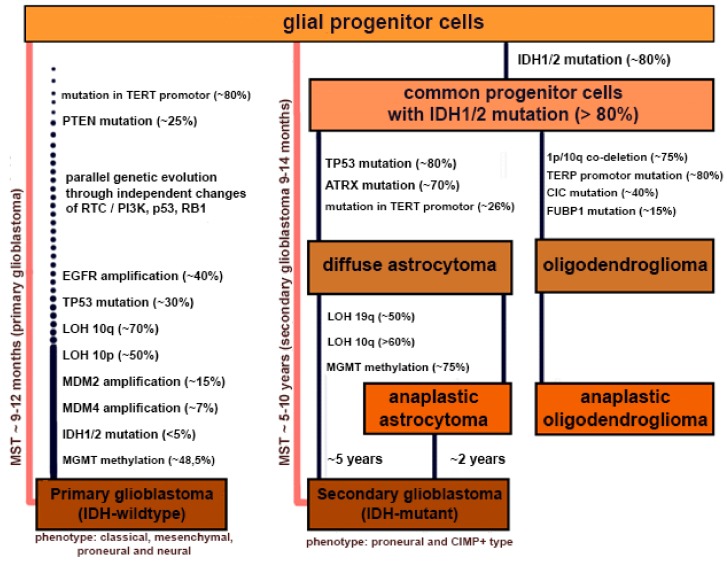
Molecular alterations responsible for glioblastoma carcinogenesis [66,67,68,69,70,71,72,73,74,75,76,77,78,79,80,81,82,83,84,85].

Histologically indistinguishable grade IV gliomas, affecting heterogeneous age groups, are a consequence of the accumulation of several genetic mutations affecting tumor development [66]. In accordance with the assessment of defined genetic parameters, GBM can be classified into primary or secondary. Approximately 90% of all cases of GBM are primary and occur in elderly patients (Table 1), in which tumor progression is more rapid due to the higher accumulation of gene mutations compared with young individuals. Generally, patients affected by primary GBM experience complication and consequently die 9–12 months after the diagnosis. In contrast, secondary GBMs develop from primary astrocytomas, bearing a lower degree of malignancy and typical of younger patients (<45 years). At the same time, there is a gradual increase in the rate of tumor cells’ proliferation, angiogenesis, drug resistance, and other parameters, which leads to an increased severity [66] (Figure 1).

An important aspect that should be taken into account in the diagnosis of GBM is its high internal heterogeneity, which is characteristic of both newly detected and recurrent tumors [86,87]. Evaluation of the internal heterogeneity of GBM, using imaging techniques such as MRI, may give important prognostic information [88,89]. The ability of dividing into subgroups GBM tumor cells within the same tumor mass, based on their spatial and temporal variability, needs to be developed [90]. The heterogeneous nature of this tumor makes it difficult to identify and validate potential biomarkers [91]. For example, morphologically different glioblastoma cells exhibit different in vitro invasion, as well as cell migration abilities, depending on the nature of the surrounding microenvironment [92]. Furthermore, the complex crosstalk between tumor cells and the microenvironment may enhance tumor growth and reduce the chances of successful drug therapy [5,93,94]. In several tumors, including glioblastoma, it was demonstrated that the alteration of the extracellular matrix (ECM) composition and the over-expression of proteolytic enzymes, due to genetic and epigenetic modifications, are responsible for a more aggressive tumor phenotype and a worse prognosis [95,96,97,98,99,100].

It is assumed that many of the invasive signs of gliomas depend on their pathological metabolism, which may either promote tumor cells invasion or create an environment in which glioma cells might gain growth advantage over normal cells [101,102].

## 2. Perspective Biomarkers

An ideal tumor marker should be easily accessible for analysis, be detected by the simplest analytical method, and be able to provide accurate information about both the presence of the disease and its severity. The ideal marker should have 100% sensitivity and specificity, sufficient half-life for detection, the ability to dynamically reflect the tumor load, and its analysis should be economically acceptable for introduction into routine practice [103,104]. However, clinical biomarkers and their respective analysis do not have to be “ideal” in order to be clinically useful for diagnostic purposes. Glioblastoma is usually clinically characterized and diagnosed with diverse physico-chemical analyses, through the use of both tissue and circulating biomarkers [3,105,106].

However, most biomarkers lack either sensitivity or specificity. Several studies have proven that screening for certain nucleic acids may have higher specificity in glioblastomas, compared with individual proteins analysis. In the same manner, low-molecular weight metabolites and lipids have shown a low specificity for systemic diagnostic tasks [3,107,108,109]. However, conflicting results were generated on this matter.

Glioblastoma characterization based on tumor genetic properties is widely accepted. In fact, since 2016, brain tumors, including glioblastomas, are internationally classified based on their molecular genetic properties, as well as the histological features linked with these properties [7].

In general, a series of mutations of DNA and de-regulation of non-coding RNA have been characterized for gliomas; the frequency of their occurrence is different and correlates with the type of brain tumor [110]. Evaluation of genetic mutations in glioma cells by genotyping circulating tumor nucleic acids allows the classification of specific tumors and the definition of the prognosis and tumor burden. Importantly, circulating tumor nucleic acid analysis enables the selection and evaluation of patients’ therapeutic efficacy window [7,111].

Currently, the assessment of genetic parameters in biopsy specimens from glioma patients is of key importance for the formulation of a refined diagnosis and the choice of the best treatment strategy [7,112]. In addition, circulating nucleic acids found in blood and other biological fluids, either free or associated with extracellular vesicles, might be used as markers for brain tumors’ early diagnosis and classification [31,111]. In particular, a number of clinically significant glioma genetic biomarkers are currently analyzed as routine practice. Among these biomarkers, the most representative are: IDH1/2 mutation status, MGMT promoter methylation, 1p/19q co-deletion, and ATRX loss [7,75,113,114]. Widely diffused molecular methods for the analysis of these genetic biomarkers and the identification of nucleic acid mutations include: Direct sequencing, high-resolution melting (HRM), immunohistochemistry, droplet digital PCR (ddPCR), and several others [110,115,116,117]. Through the use of these advanced techniques, it is nowadays possible to classify histologically indistinguishable GBM by the presence/absence of genetic mutations, which has an important therapeutic, prognostic, and experimental value [64,118]. The issues with the analysis of nucleic acids in peripheral blood are similar with the problems encountered when searching for high-molecular polar central nervous system (CNS)-derived compounds. In particular, low concentration, low abundance of the compounds in the systemic circulation, coupled with their weak penetration thought the BBB, make brain tumor-derived nucleic acids difficult to be identified as circulating molecules [31,119,120,121,122,123,124].

### 2.1. microRNAs (miRNAs)

In the last decade, a growing body of evidence has shown that a class of small non-coding RNAs (ncRNAs), called miRNAs, are involved in several physiological and pathological processes. Specifically, miRNAs are ncRNAs 20–22 nucleotides long able to modulate the expression of specific genes by degrading the corresponding mRNAs or by blocking their access to the ribosomal machinery. Thanks to the advancement of new high throughput technologies, a great amount of molecular data regarding the expression profile of miRNAs in several cancers has been generated [125]. Furthermore, through the development of innovative prediction bioinformatics tools, specific sets of miRNAs have been found modulated in a wide variety of tumors, including brain cancers [126,127,128,129,130,131]. Different research groups have identified specific miRNAs associated to glioma and glioblastoma diagnosis and prognosis. Recently, Jesionek-Kupnicka and co-workers validated a panel of five miRNAs—miR-21, miR-125b, miR-34a, miR-181d, and miR-648—strictly involved in MGMT and TP53 alterations, therefore responsible for the progression of glioblastoma [132]. Among these miRNAs, miR-21 and miR-181d were also found de-regulated in other studies, suggesting their potential involvement in glioblastoma carcinogenesis [133,134,135,136]. Additionally, miR-144 and miR-29a were also associated with glioblastoma development [137,138,139]. Although many data have been generated on this matter, further validation studies are needed in a wide cohort of glioblastoma patients in order to confirm the diagnostic and prognostic significances of these miRNAs.

Although evaluation of miRNA expression levels in patients represents an innovative strategy for the early detection of various diseases, including tumors, even today, miRNAs cannot be used yet as reliable cancer diagnostic markers, given their low specificity and selectivity. In fact, the main disadvantage of the use of miRNAs as biomarkers is related with the fact that their modulation is often involved in different physiological and pathological conditions (e.g., chronic inflammation or other non-tumor pathologies) [140]. Additionally, although involved in glioblastoma development, the aforementioned miRNAs were found to be able to modulate a higher number of genes involved in several different processes at the same time. Therefore, the use of miRNAs as biomarkers needs to be coupled with an accurate clinical evaluation of the patient and other molecular analyses which are essential for a correct diagnosis of glioblastoma.

### 2.2. Proteins

Proteins can be used as diagnostic and prognostic markers in patients with brain tumor. They can be detected both in glioblastoma tissues [120] and in liquid matrices: blood and its derivatives [107,141,142], cerebrospinal fluid (CSF) [108], and urine [69]. The main approach currently used for searching for glioblastoma multiforme protein tumor markers is the study of the proteomic profile (bottom-up or top-down) [66] coupled with gene expression study [72,143]. Importantly, single tumor cell protein expression profiling does not provide a complete picture of the proteomic profile for the whole tumor bulk. The comparison of full proteomic tumor profiles takes into account both the nature and frequency of post-translational changes during cancer development, and allows to fully characterize the object of study. In brain tumors, including glioblastomas, differences in post-translational changes between pathological and normal cells populating the nervous system have been reported. It should also be noted that the study of protein post-translational modifications in GBM contributes to the search for new markers and therapeutic approaches to treat this disease [144,145].

Currently, many proteins have been characterized and changes in their qualitative or quantitative composition are currently associated with tumor progression in cancer patients [146,147]. Several glioblastoma protein markers have been reported in literature. Among these proteins, the most representative are: VEGF and angiogenesis-associated proteins (FGF-b; IGFBP-2; Ang2; EGF and others), extracellular matrix proteins (TSP1/2; TNC; Cyr61/CCN1; OPN, etc.), matrix metalloproteinases (MMP-2; MMP-9; AEG-1), cell line associated proteins (GFAP), macrophage migration inhibitory factor (MIF), and functionally-related proteins (DD-T; CD74, CD44, CXCR2 and CXCR4) [148,149,150]. Additionally, embryonic antigens and other proteins can be used for the diagnosis and the prognostic evaluation of glioblastoma development [108,120,151,152,153]. Many of these proteins are used for the diagnosis of other tumors [114]. However, other proteins, as for example VEGF, are low-specific, since a change in their expression level is observed not only during brain tumors development, but also in other oncological as well as non-tumoral conditions [154,155,156,157]. In order to avoid false-negative and false-positive results due to the lack of specificity of protein markers, nowadays the better approach is performing an integrated multi-parametric evaluation, based on the qualitative and/or quantitative analysis of several different protein markers at the same time [66,108]. The advantages of multi-parametric evaluation include the possibility of implementing this approach based on the existing scientific and technical base and the high differentiating ability of this method, which allows, in addition to binary analysis (healthy/ill), to diagnose the specific tumor subtype [108,151,158]. A disadvantage in the identification of tumor-derived proteins in patients’ biological fluids, such as blood serum or CSF, can be their low concentration and their relative abundance compared with other proteins present in the blood serum (matrix effect). To increase the sensitivity, concentrating patients’ samples might be necessary. Moreover, to ensure a higher specificity of the detection methods used, extensive validation of the selected methodology is further needed [1,152].

### 2.3. Small Molecules

The identification of changes in concentrations of small molecules or low-molecular compounds in glioblastoma cells, compared with normal cells, can be used as diagnostic and prognostic markers, as well as for a correct classification of glioblastoma [159,160]. The category of small molecules includes cell lipids, metabolites, organic compound, and monomers able to rapidly diffuse across cell membranes, thus reaching intracellular and extracellular spaces. However, given their low specificity and low molecular weight, their use as a marker is little informative. Therefore, they can be successfully used only to refine the diagnosis when coupled with more specific methods such as MRI [105].

Despite the fact that small molecule metabolites have low diagnostic potential for systemic diagnosis, this class of substances is the most promising as rapid diagnostics tools in the context of navigation surgery. The use of mass spectrometry methods helps to successfully define the brain tumor boundaries, the surgical field, and the exploration of the postoperative cavity, both ex vivo and in real-time [159,161].

### 2.4. Circulating Tumor Cells (CTCs)

Circulating tumor cells are separated from the primary tumor or metastasis and enter the systemic circulation. Approaches for the detection of circulating tumor cells include the use of antibodies for immunohistochemical evaluation and subsequent genetic analysis [162], visual methods for research [73], and evaluation of cell sizes [70]. The mechanism of penetration of circulating brain-derived tumor cells into the bloodstream is not fully understood; however, it is assumed that the glioma tumor cells penetrate into the bloodstream due to great invasiveness, which helps to overcome the BBB or even disrupt the BBB function during tumor development [70,163].

The detection of circulating tumor cells in patients’ blood samples has a significant clinical potential in CNS malignant neoplasms, thanks to the advantage of performing disease diagnosis and prognosis using blood specimens without any invasive and risky neurosurgical procedure [164,165,166].

Even though GBM tends to be a cranial-restricted tumor with distant metastases accounting 0.5–2% of all GBM patients, several research groups have demonstrated the isolation of CTCs from GBM cells, both in vivo and in vitro, with a variety of methods (glial fibrillary acidic protein-GFAP-based assay, immunomagnetic and immunofluorescence-based cell selection) [164,167,168]. Nonetheless, more researches are needed in this area, since it is not clear yet whether CTCs are actually able to explain GBM behavior, its extremely low percentage of metastases, or if they are truly indicative, at least of the majority, of the tumor genetic aberrations [104].

### 2.5. Extracellular Vesicles

Extracellular vesicles are naturally occurring cellular products composed of an outer hydrophobic lipid bilayer surrounding a hydrophilic aqueous core. Varying in diameter they can be categorized as exosomes (30–100 nm), microvesicles (50 nm–1.5 μm), oncosomes (100–400 nm), apoptotic bodies (50 nm–2 μm), and large oncosomes (1–10 μm). They serve as transporting vehicles for lipids, proteins, genetic elements, and various soluble intracellular material. Their role is to regulate the homeostasis of cell microenvironment by regulating neighboring cells and transducing them specific functional messages. It is well documented that extracellular vesicles are closely related to cancer progression by mediating the transportation of special factors able to control and deregulate proliferation, drug resistance, migration, angiogenesis induction, and invasion. It is proved that glioma cells are capable of producing all types of extracellular vesicles [169].

In particular, glioblastoma cells secrete extracellular vesicles, the molecular composition of which reflects the characteristics of the parental cells, which makes extracellular vesicles a promising object of analysis for diagnostic purposes [31]. Glioma cells are capable of producing several types of extracellular vesicles such as exosomes, microvesicles, and apoptotic bodies, as well as oncosomes, an atypically large type of vesicle produced selectively by tumor cells [170]. Extracellular vesicles are able to overcome the BBB, both in physiological and pathological conditions. They can be found in the bloodstream, as well as in CSF [15,171]. Modern theories of oncogenesis consider extracellular vesicles as a mechanism of intercellular communication, allowing tumor cells to acquire various properties necessary for tumor development [170]. Moreover, tumor-derived extracellular vesicles bear the ability to selectively suppress the host’s immune response and to sustain tumor carcinogenesis [15,172].

Extracellular vesicles, as well as circulating tumor cells, are carriers for oncogenic growth factors, receptors, enzymes, transcription factors, signaling and immunomodulating molecules, DNA of mutated and non-mutant oncogenes, RNA (including non-coding RNA, miRNA, and retrotransposons), proteins, lipids, and metabolites whose composition reflects the cell of origin, which makes them excellent biomarker reservoirs [162].

Finally, some of the main biomarkers identified in extracellular vesicles and linked with GDM exosomes is the glioma-specific receptor of epidermal growth factor (EGFRvIII), which is a piece of mRNA with the receptor (found in serum), miR21 which is an essential miRNA for GBM diagnosis (found in serum), and the mutant IDH1 mRNA found in CSF [173,174,175,176].

Overall, the above paragraphs showed that several biomarkers from different sources and of different nature were identified for glioblastoma diagnosis and prognosis (Table 2). However, there are still no concordant guideline about the use of single or multiple biomarkers evaluation in clinical setting.

## 3. Other Biological Specimens

Glioblastoma multiforme can be further evaluated using physicochemical analysis, in particular mass spectrometry methods, both in tumor tissue samples and biological fluids (blood, CSF, or urine).

### 3.1. Tumor Tissues

Glioma tissue samples can be precisely characterized through the identification of tumor-specific protein, lipid, genetic, or metabolic molecules for diagnostic or navigational surgery [106,108,159,176,178]. However, the biopsy procedure itself can have harmful effects for patients, as even moderate hemorrhage might occur during the procedure, leading to neurological function impairment or even life-threatening effects [179]. However, molecular analysis of glioma tissues with intraoperative mass spectrometry might overcome the above listed issues, allowing a safer intraoperative diagnosis, characterized by a quicker and more accurate identification and characterization of the lesion [71,180]. Existing models built on protein analysis, such as PROTGLIO, demonstrate higher accuracy in predicting the course of the disease through tumor tissues analysis [91]. The formulation of such a diagnosis is based on the qualitative and/or quantitative assessment of tumor-specific molecules and oncometabolites [181,182].

For example, glioma cells’ lipid profile compared with the neighboring normal brain cells’ one, demonstrates significant qualitative and quantitative differences. Intraoperative diagnosis of gliomas by using rapid detection tools for low-molecular tumor-cell components are well studied and can be introduced into clinical practice; however, the value of their practical application, especially in relation to the surgical treatment of GBM, is significantly reduced by brain tumor invasiveness [31,33]. Although this difference in biomolecules produced selectively by brain tumor cells cannot be used as a diagnostic biomarker since it lacks of specificity [183,184], it can be used intraoperatively to increase the efficacy of surgical intervention [106]. For example, Pirro et al. demonstrated the efficiency of the electrospray desorption ionization method (DESI-MS) as an intraoperative tool to accurately assess brain tumor boundaries. The degree of tumor infiltration into the white or gray matter of the brain was assessed by detecting the signal of *N*-acetylaspartate and membrane lipid complexes, while the mutation status of tumor isocitrate dehydrogenase was assessed by detecting 2-hydroxyglutarate [109]. Low-molecular tumor markers are the most promising candidates as local intraoperative markers, both to precisely define tumor boundaries and to improve navigation surgery [185,186].

Moreover, the identification and quantification of metabolic products such as trimetilamin-*N*-oxide, *N*-atsetilputrestsin, and uridine by mass spectrometry helps to identify key differences between healthy and tumor tissue, making a step ahead for a correct tumor staging and a fast identification of IDH mutations [187,188].

### 3.2. Biological Fluids

A promising approach for glioma diagnosis comes from the study of biological fluids using liquid biopsy. Compared with conventional tissue sampling, liquid biopsy is minimally invasive, safe, and reproducible. Due to the heterogeneity of glioblastomas, circulating markers, such as extracellular DNA, might be more representative of the entire tumor cellular population compared with classical tumor biopsies [189,190]. Common limitations of liquid biopsy are: the high variability of the genetic mutations encountered, low concentration and low representation of target analytes. The main circulating biomarkers found in biological fluids from GBM patients are circulating tumor cells [117], extracellular vesicles (microvesicles and exosomes) [191,192], circulating tumor nucleic acids [193,194,195,196], and tumor-specific proteins [197,198,199,200].

The fact that the CSF is in direct contact with brain tumor cells and the significantly lower background level of matrix substances, primarily cerebrospinal proteins, makes it a potentially valuable patient specimen for specific gliomas biomarkers identification [119,146,201]. Based on cerebrospinal protein’s proteomic profile, a diagnostic model (fingerprint diagnostic model) was built to identify the profile of glioma-specific cerebrospinal proteins. This experimental model showed high sensitivity and specificity for both diagnosis and differentiation of glioma from extra-cerebral tumor processes and benign brain tumors [108]. Also, tumor-specific metabolites and circulating tumor nucleic acids could be detected in the spinal fluid. As a limitation, both the complexity and risks linked with lumbar puncture for CSF collection make the technique difficult to use for early glioma diagnosis [202,203].

Plasma is the most promising biological specimen currently used for the early diagnosis of gliomas. In peripheral blood, several markers—such as tumor metabolites [204], circulating extracellular nucleic acids [120], circulating cancer cells [124,205], proteins associated with the tumor development [107,141,142], and extracellular vesicles [172]—can be easily detected through the use of new highly-sensitive molecular technologies, including mass spectrometry, high-sensitive ELISA assay, ddPCR, etc. The use of these highly-sensitive technologies is necessary because in plasma, the concentration of brain tumor-specific markers, including proteins, might be greatly reduced to ng/mL or even lower concentrations, thus at the limit of detection [206]. Miyauchi E et al., demonstrated the effectiveness of an information-independent analysis using high-resolution mass spectrometry, where plasma concentration of potential tumor marker proteins in glioma patients was successfully detected, and significantly different in their expression levels when compared with control samples [151].

Similarly, like CSF, urine is also able to accumulate metabolic products, thus reflecting their systemic level changes. Due to these qualities, urine represents a promising source of tumor biomarkers. Experimental models have demonstrated detectable levels of C6 glioma-derived proteins in the urine of tumor-bearing mice compared with their controls. These experiments have shown that a subclass of proteins in urine can reliably identify the presence of brain tumor. The described experimental approach is highly sensitive and allows the detection of glioblastoma before the onset of MRI-detectable brain tissue changes [207].

As previously mentioned, the evaluation of MMP-9/NGAL complex in urine samples of glioblastoma patients also positively correlated with MRI-based tumor assessment [208]. Accordingly, Wu and co-authors, using quantitative LC-MS/MS proteome assessment, detected 27 tumor-derived proteins in urine, whose levels were significantly modulated following tumor resection [66]. Additionally, low molecular weight metabolites deriving from the brain tumor bulk might be found at a detectable concentration in patients’ urine samples. Such an analysis is not specific enough for an unambiguous diagnosis, but it may be useful for more precise diagnosis when used as additional methodology together with other diagnostic tools or for routine monitoring of postoperative patients [87]. Despite the fact that many potential biomarkers have been successfully detected in urine in tumor animal models, its successful application in clinical practice requires additional studies and extensive methods validation [66].

## 4. Discussion

In the last century, a constant development of new pharmacological strategies for cancer treatments has been observed. However, despite the efficacy of these new treatments, the mortality rates for aggressive tumors is not decreasing significantly yet because increased incidence rates couples with a rise in drug resistance mechanisms [209]. Due to the absence of targeted medical therapy and the ineffectiveness of surgical intervention for the treatment of glioblastoma, the task of developing methods for the early diagnosis of this disease is of particular importance. Existing clinical approaches for the detection of glioblastoma are often ineffective and strictly dependent on results obtained using neuroimaging methods, such as MRI. However, the development of brain tumors, especially in the early stages, may not have clear and early clinical symptoms. Moreover, the use of neuroimaging methods is hardly applicable for mass screening, and such an assessment does not always allow to effectively determine the presence and the malignancy of a brain tumor [18,19].

The selection of promising biomarkers is also associated with the choice of a robust analytical method for their analysis. Mass spectrometry applied to the analysis of biological materials has several advantages, including sensitive detection, high performance, selectivity, and the possibility to analyze a wide range of chemical compounds. In particular, high-performance mass spectrometry is important for the analysis of biological samples with intrinsic variability, such as tumor tissue. In the long run, mass spectrometry methods can replace a significant number of laboratory diagnostic methods. Compared to methods based on the use of antibodies, mass spectrometry detection methods have been demonstrated to be the best indicators, with an improved detection limit, reproducibility, accuracy, and precision [210].

In order to accomplish early diagnosis of glioblastoma, the most promising biomarkers are tumor-derived proteins and nucleic acids. The analysis of nucleic acids today is introduced into clinical practice and is currently used for diagnosis and prognosis of the disease. However, nucleic acid analysis alone is not sufficient to effectively define the nature of the tumor. On these bases, further studies using other analytical approaches and other types of samples are necessary to ameliorate diagnosis of patients.

In this context, proteomic analysis using mass spectrometry methods offers promising results, allowing the detection of both tumor-specific proteins and post-translational modifications. The qualitative or quantitative evaluation of protein expression pattern or protein post-translational modification changes may serve as a CNS malignant tumor marker [108,211]. Proteomic analysis using mass spectrometry allows the researcher not only to compare the complete proteomic profiles of healthy and pathological samples, but also to correlate the presence of a set of protein alterations to the presence of a specific disease state [151]. This aspect is extremely important for the introduction of mass spectrometry into the routine clinical diagnosis of CNS oncological diseases. It should also be noted that the technical characteristics of modern mass analyzers allow the analysis of several individual molecular patterns, which are characteristic of several pathologies during one analytical cycle, thus providing prospects for the introduction of this method of analysis into routine practice. The use of multiparameter analysis will help the creation of a database of tumor-specific markers for clinical diagnosis by mass spectrometry. Experimental modeling of diffuse gliomas is a difficult task, since well-known preclinical models, including in vitro cell lines and xenografts, do not reliably reproduce some of the aspects of glioma biology [212]. However, experimental cell models and xenografts are extremely useful for testing and validating biological samples using mass spectrometry methods.

For intracranial tumors, which are unavailable for frequent tissue biopsy, biological fluids are the preferred source for monitoring the levels of tumor biomarkers. Liquid biopsy has a number of significant advantages over tissue biopsy and it is the most promising high-throughput clinical screening method. Differential diagnosis of gliomas demonstrates high accuracy and specificity based on the proteomic profiling studies in CSF. However, risks associated with CSF lumbar sampling might prevent the routine introduction of this methodology in clinical practice. In contrast, patient-derived blood- and urine-based liquid biopsies represent promising non-invasive methods for glioblastoma, early diagnosis, staging which need to be developed further given their high-throughput and high-compliance potentials.

## 5. Conclusions

Further study on genetic, protein, and metabolic changes occurring during both glioma and glioblastoma multiforme development is necessary to improve early diagnostic methods, as well as to develop novel personalized anti-cancer therapies. Currently, a significant number of potential tumor-specific markers of different chemical natures have been identified, whose changes in the qualitative and quantitative composition are associated with glioblastoma multiforme progression. However, only a few of these identified markers have found application in clinical practice as prognostic or diagnostic markers, none of which are used yet as a method of initial diagnosis [7].

In addition to circulating nucleic acid mutations detection, mass spectrometric-based multiparametric analysis of circulating tumor-associated proteins is the most promising approach potentially used in clinical practice [66]. The advantage of both approaches is their relatively high specificity, which allows to establish the status of the patient (healthy/ill), to discriminate the neoplastic transformation from other processes (including CNS inflammation, neuro-degeneration, etc.), and, finally, to establish the subtype of the tumor [7,108,202]. Therefore, proteomic analysis based on the use of mass spectrometric methods is a powerful tool for studying the biology of glioblastoma multiforme and developing clinical methods for early diagnosis.

## Figures and Tables

**Table 1:** Characteristics of primary and secondary glioblastoma [71,72,73,74,75,76,77,78,79,80,81,82,83,84,85].

Status/Feature	Primary Glioblastoma	Secondary Glioblastoma
Positive status mutation of IDH gene	<5%	~80%
Preceding cancer disease	Not identified; detected for the first time (de novo)	Diffuse astrocytoma; anaplastic astrocytoma
The percentage of all detected glioblastoma	90%	<10%
The average age of diagnosis	62	44
Sex ratio (M:W)	1.42:1	1.05:1
Median overall survival		
surgical treatment and radiotherapy	9.9 months	24 months
surgical treatment, radiotherapy and chemotherapy	15 months	31 months
Localization	Supratentorial	Predominantly frontal
Necrosis	Extensive	Limited
TERT promoter mutation	72%	26%
TP53 mutation	27%	81%
ATRX mutation	Rarely	71%
EGFR mutation	35%	Rarely
PTEN mutation	25%	Rarely

**Table 2:** Most used and studied biomarkers for glioblastoma.

Relevant Biomarkers for Glioblastoma				
Molecule	Specimen	Detection Methodology	Usage	Reference
IDH1/2 mutation	Frozen tissue, Formalin-fixed paraffin-embedded tissue	Direct sequencing, high-resolution melting (HRM), immunohistochemistry, and droplet digital PCR (ddPCR)	Diagnosis, prognosis, prediction	Louis et al. [7], Hegi et al. 2005 [113], Hegi et al. 2008 [114], Okita et al. [75]
Methylated MGMT promoter				
1p/19q co-deletion				
ATRX deletion				
miR-21, miR-125b, miR-34a, miR-181d, and miR-648	Formalin-fixed paraffin-embedded tissue	miRNeasy FFPE Kit, reverse transcriptase PCR	Diagnosis, prognosis	Jesionek-Kupnicka et al. [132]
miR-144 and miR-29	Frozen tissue, Formalin-fixed paraffin-embedded glioma tissue	DNA/RNA/miRNA Universal kit, miRCURY Isolation Kit, Quantitative real time PCR	Prognosis, prediction	Cardoso et al. [137] Yang et al. [138] Zhao et al. [139]
VEGF, FGF-b, IGFBP-2, Ang2, and EGF	Frozen tissue, whole blood, CSF	SWATH mass spectrometry, quantitative targeted absolute proteomics	Prognosis, prediction	Mammana et al. [148], Mangano et al. [149], Presti et al. [150], Miyauchi et al. [151], Ludwig et al. [152]
TSP1/2, TNC, Cyr61/CCN1, and OPN				
MMP-2, MMP-9, and AEG-1				
GFAP				
Migration inhibitory factor (MIF)				
DD-T; CD74, CD44, CXCR2 and CXCR4				
Low-molecular compounds (lipids and oncometabolites)	Frozen tissue	MALDI-TOF mass spectrometry, metabolomic profiling	Diagnosis, prognosis	Longuespée et al. [159], Mörén et al. [160]
Circulating tumor cells (CTCs)	Whole Blood	Glial fibrillary acidic protein (GFAP)-based assay, immunomagnetic and immunofluorescence-based cell selection	Prognosis, prediction	Macarthur et al. [164], Müller et al. [177], Sullivan et al. [168]
Exosomes (EGFRvIII, miR21, mutant IDH1 mRNA)	Serum, CSF	BEAMing and droplet digital PCR	Diagnosis, prognosis	Al-Nedawi et al. [173], Akers et al. [174], Chen et al. [175]
